# The Association between Left Ventricular End-Diastolic Diameter and Long-Term Mortality in Patients with Coronary Artery Disease

**DOI:** 10.31083/j.rcm2403084

**Published:** 2023-03-08

**Authors:** Qiang Li, Haozhang Huang, Xiaozhao Lu, Yajuan Yang, Yunhan Zhang, Weihua Chen, Wenguang Lai, Guoxiao Liang, Shanshan Shi, Xiaoyan Wang, Jiyan Chen, Shiqun Chen, Xiaoming Yan

**Affiliations:** ^1^Department of Cardiology, Guangdong Cardiovascular Institute, Guangdong Provincial People’s Hospital, Guangdong Academy of Medical Sciences, 510080 Guangzhou, Guangdong, China; ^2^Department of Guangdong Provincial Key Laboratory of Coronary Heart Disease Prevention, Guangdong Cardiovascular Institute, Guangdong Provincial People’s Hospital, Guangdong Academy of Medical Sciences, 510080 Guangzhou, Guangdong, China; ^3^Department of Cardiology, The First People’s Hospital of Foshan, 528000 Foshan, Guangdong, China; ^4^Department of Ultrasound Medicine, Yunnan Fuwai Cardiovascular Hospital, Kunming Medical University, 650500 Kunming, Yunnan, China; ^5^Department of Cardiology, The School of Clinical Medicine, Fujian Medical University, 364000 Longyan, Fujian, China; ^6^Department of Nuclear Medicine, the First Affiliated Hospital of Sun Yat-sen University, 510080 Guangzhou, Guangdong, China; ^7^Department of Information Technology, Guangdong Provincial People’s Hospital, Guangdong Academy of Medical Sciences, 510080 Guangzhou, Guangdong, China

**Keywords:** mortality, prognosis, coronary artery disease, left ventricular, left ventricular end-diastolic diameter

## Abstract

**Background:**

Left ventricular end-diastolic diameter (LVEDD) is a common parameter in echocardiography. Increased LVEDD is associated with left ventricular (LV) dysfunction. However, the association between LVEDD and all-cause mortality in patients with coronary artery disease (CAD) is uncertain.

**Methods:**

This study enrolled 33,147 patients with CAD who had undergone transthoracic echocardiography between January 2007 and December 2018 from the Cardiorenal Improvement study (NCT04407936). The patients were stratified into four groups based on the quartile of LVEDD (Quartile 1: LVEDD ≤43 mm, Quartile 2: 43 mm < LVEDD ≤46 mm, Quartile 3: 46 mm < LVEDD ≤51 mm, Quartile 4: LVEDD >51 mm) and were categorized into two groups (Quartile 1–3 versus Quartile 4). Survival curves were generated with the Kaplan-Meier analysis, and the differences between groups were assessed by log-rank test. Restricted cubic splines and cox proportional hazards models were used to investigate the association with LVEDD and all-cause mortality.

**Results:**

A total of 33,147 patients (average age: 63.0 ± 10.6 years; 24.0% female) were included in the final analysis. In the average follow-up period of 5.2 years, a total of 4288 patients died. The mortality of the larger LVEDD group (Quartile 4) was significantly higher than the lower LVEDD groups (Quartile 1–3) (18.05% vs 11.15%, *p <* 0.001). After adjusting for confounding factors, patients with the larger LVEDD (Quartile 4) had a 1.19-fold risk for all-cause mortality (95% CI: 1.09–1.30) compared with the lower quartile (Quartile 1–3).

**Conclusions:**

Enlarged LVEDD is an independent predictor of all-cause mortality in patients with CAD. LVEDD measurements may be helpful for risk stratification and providing therapeutic targets for the management of CAD patients.

## 1. Introduction

Left ventricular end-diastolic diameter (LVEDD) derived from echocardiography is an important parameter of cardiac chamber size and left ventricular (LV) function. It is an easily measured non-invasive technique and is widely used in clinical practice.

Alterations in cardiac structure and function caused by myocardial remodeling are common in patients with cardiovascular diseases. Myocardial remodeling is defined as a change in heart size, shape and function following a heart injury, driven by multifactorial processes including changes in genome, molecular, cellular and interstitial function [1]. The volume overload produced by mitral regurgitation or aortic stenosis results in compensatory LV dilation [2, 3]. Similarly, the adaptation of LV systolic dysfunction in patients with other heart diseases results in LV dilation [4, 5].

Echocardiography is often used to measure and quantify the severity of ventricular remodeling and cardiac structural abnormalities, of which LVEDD is one of the most important indicators. Previous studies have shown that LVEDD is associated with cardiovascular events and all-cause mortality. Kitaoka H *et al*. [6] found that in patients with dilated cardiomyopathy or heart failure (HF), LV dilatation was significantly associated with increased mortality [6, 7, 8]. In patients with hypertrophic cardiomyopathy (HCM), LVEDD was also a significant predictor of mortality [9].

However, the prognostic value of LVEDD in patients with coronary artery disease (CAD) is uncertain [10]. Therefore, we sought to investigate the association between LVEDD and long-term all-cause mortality in CAD patients.

## 2. Materials and Methods

### 2.1 Study Design and Data Collection

**Fig. 1.**
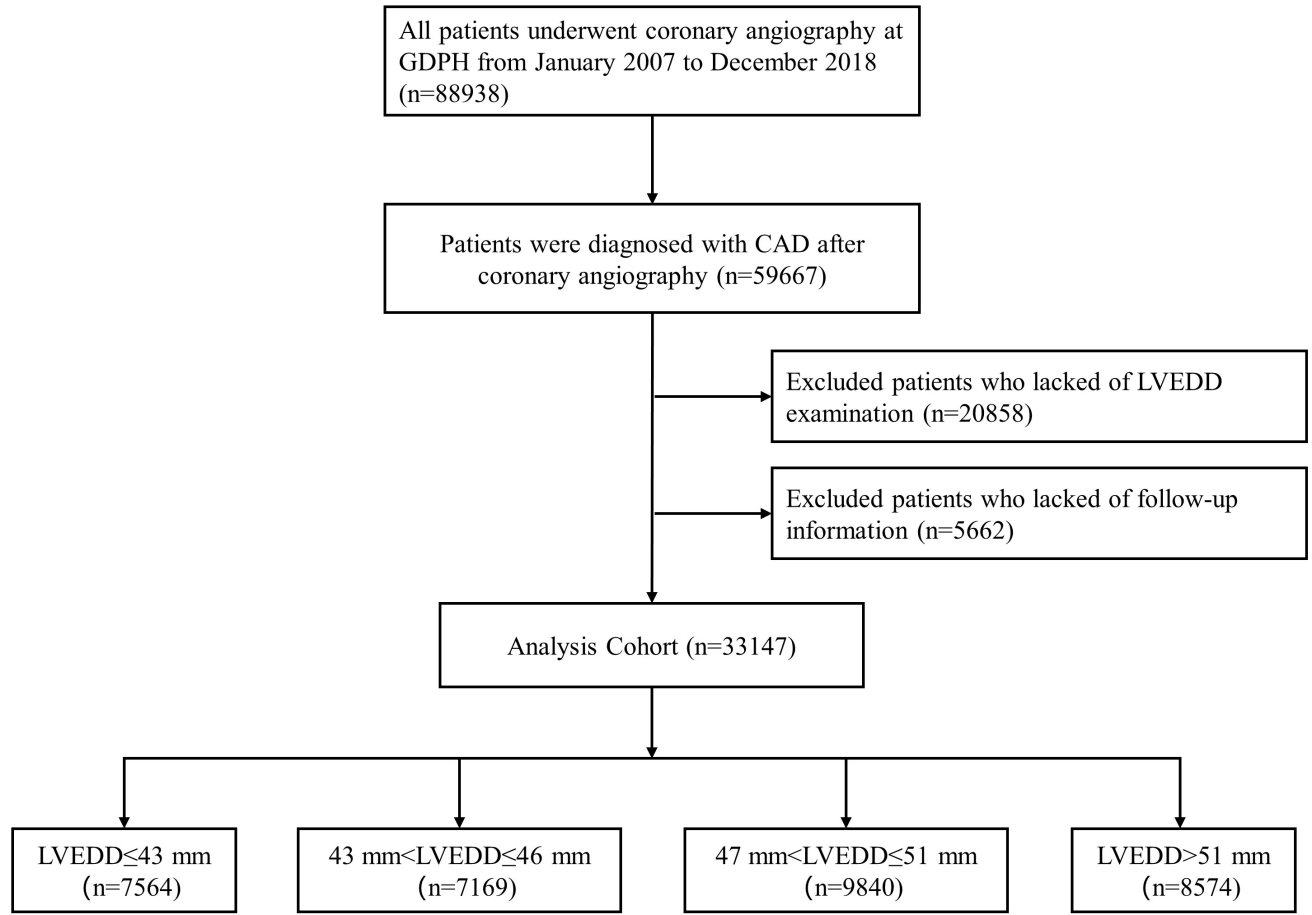
**The flowchart of enrolled patients of the study**. CAD, Coronary Artery Disease; GDPH, Guangdong Provincial People’s Hospital in China; LVEDD, left ventricular end-diastolic diameter.

The study data was obtained from the registry of the Cardiorenal Improvement (CIN) study (ClinicalTrials.gov NCT04407936) during January 2007 and December 2018. This was a single-center, observational, retrospective cohort study from the Guangdong Provincial People’s Hospital in China. A total of 33,147 CAD patients were included in the final analysis after excluding patients who did not undergo LVEDD examination or lacked follow-up data (Fig. 1). This study adhered to guidelines of the Declaration of Helsinki and was approved by the Research Ethics Committee of Guangdong Provincial People’s Hospital (No. GDREC2019555H).

This study data source was the Clinical Management System of the Guangdong Provincial People’s Hospital’s electronic health record which contained detailed demographic characteristics, medical histories, laboratory tests, medications and other clinical data. The information on death events and the date of death for each patient were retrieved from Guangdong Public Security System, which was linked to CIN dataset by unique identified numbers. The indications for coronary angiography (CAG) or percutaneous coronary intervention (PCI) were signs or symptoms of ischemia, elevated diagnostic enzymes, or abnormal electrocardiogram findings. All treatment was performed based on standard clinical practice guidelines [11, 12, 13].

### 2.2 LVEDD Measurement

**Fig. 2.**
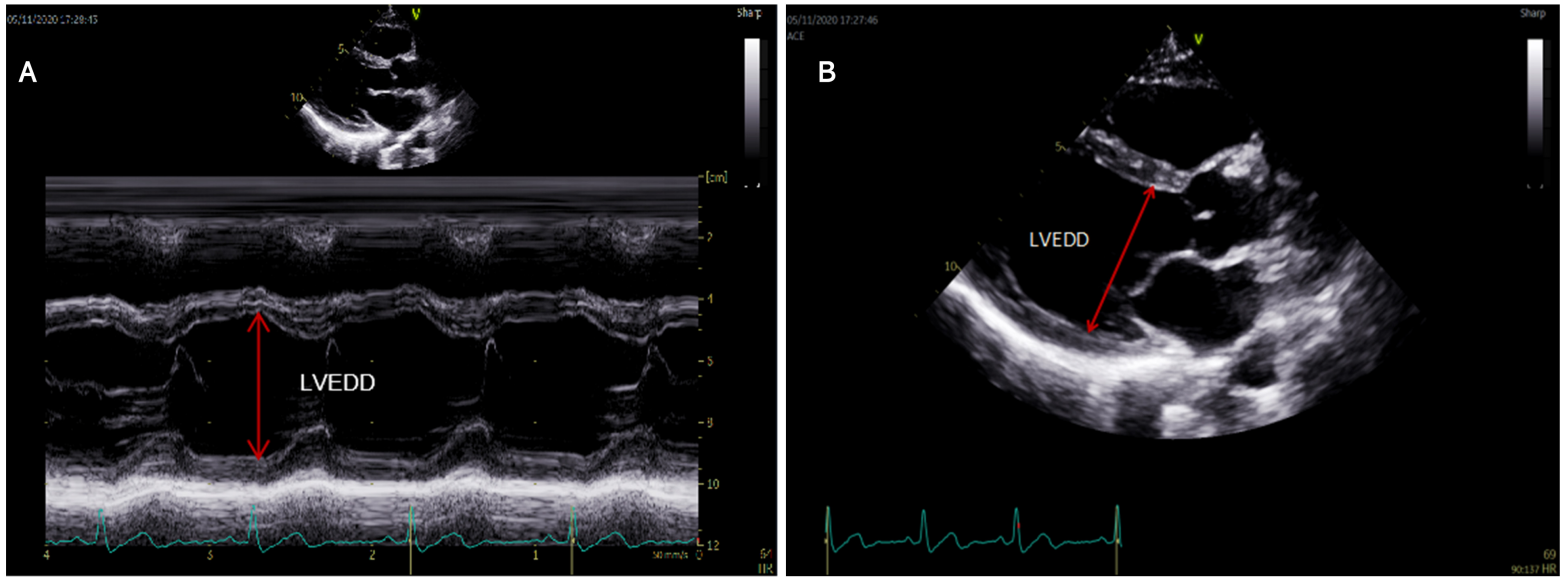
**The measurement methods of LVEDD**. (A) A motion type scan of the parasternal long-axis in two dimensional views was used to measure LVEDD. (B) In cases where the motion mode cursor could not be aligned perpendicularly to the LV long axis, LVEDD was measured directly on 2D images.

Echocardiography was performed by same team of cardiac ultrasound physicians at the timing of admission. A motion type scan of the parasternal long-axis in two-dimensional views was used to measure LVEDD (Fig. 2A). In cases where the motion mode cursor could not be aligned perpendicularly to the LV long axis, LVEDD was measured directly on 2D images (Fig. 2B) [14].

### 2.3 Study Definitions and Endpoint 

The primary endpoint was all-cause long-term mortality. CAD was confirmed by CAG and based on the 10th Revision Codes of the International Classification of Diseases (ICD-10; I20.xx–I25.xx, I50.00001 and I91.40001 *et al*., **Supplementary Table 1**). The type of disease was extracted from the electronic medical records and defined by the ICD-10 code, (i.e., Diabetes mellitus (DM) and hypertension (HT)). Relative wall thickness was calculated from the formula (2× diastolic left ventricular posterior wall thickness)/LVEDD, and was considered as left ventricular remodeling if >0.42. Estimated glomerular filtration rate (eGFR) was estimated by the Modification of Diet in Renal Disease (MDRD) formula, and chronic kidney disease (CKD) was defined as eGFR <60 mL/min/1.73 $m^{2}$ [15]. Congestive heart failure (CHF) was defined as New York Heart Association (NYHA) functional class >2, Killip class >1, or pulmonary edema.

### 2.4 Statistical Analysis 

According to the quartile of LVEDD, the patients were divided into four groups (Quartile 1: LVEDD ≤43 mm, Quartile 2: 43 mm < LVEDD ≤46 mm, Quartile 3: 46 mm < LVEDD ≤51 mm, Quartile 4: LVEDD >51 mm). At baseline, continuous variables and categorical variables were presented as mean ± standard deviation and frequency (%) respectively. The population characteristics of the different groups were compared by one-way analysis of variance (ANOVA) or Pearson chi-squared test. Kaplan–Meier methods and survival curves were plotted for prognostic analysis.

We used log-rank tests to distinguish the survival differences among different groups. The cox proportional hazards regression models (hazards ratio [HR] and 95% confidence interval [CI]) and restricted cubic splines were used to evaluate the relationship between LVEDD and all-cause mortality in CAD patients. Model 1 was unadjusted, Model 2 was adjusted for age and gender, and Model 3 was adjusted based on Model 2 adding the variables which were significant based on univariate Cox proportional hazards regression and associated with mortality according to clinical data. The subgroup analysis was conducted based on the stratification of age, HF, HT, DM, CKD, atrial fibrillation (AF), and acute myocardial infarction (AMI). All analyses were performed by R software (version 4.0.3, R Foundation for Statistical Computing, Vienna, Austria). A two-sided *p*-value < 0.05 indicated significance for all analyses.

## 3. Results

### 3.1 Clinical Characteristics

The final analysis included 33,147. The mean age was 62.9 ± 10.61 years, and 7959 (24.01%) were female. The mean LVEDD was 48.49 ± 6.90 mm. Patients were divided into four groups: Quartile 1 (LVEDD ≤43 mm, n = 7564), Quartile 2 (43 mm < LVEDD ≤46 mm, n = 7169), Quartile 3 (46 mm < LVEDD ≤51 mm, n = 9840), Quartile 4 (LVEDD >51 mm, n = 8574). In total, 9000 (27.16%) patients had DM 10,554 (33.10%) patients had anemia. 6708 (20.24%) patients had an AMI and 23,824 (71.87%) patients underwent PCI (Table 1). Compared with the lower Quartile (Q1–Q3), the largest Quartile (Q4) group was more likely to have CKD, CHF and lower left ventricular ejection fractions (LVEF). Detailed information of the patient characteristics is shown in Table 1.

**Table 1.** **Baseline characteristics**.

Characteristics		LVEDD					
		Overall	Q1	Q2	Q3	Q4	*p*-value
		(N = 33,147)	(N = 7564)	(N = 7169)	(N = 9840)	(N = 8574)	
Demographic							
	Age, years	62.95 (10.61)	64.78 (10.26)	62.89 (10.48)	62.07 (10.73)	62.39 (10.68)	<0.001
	Age >75, n (%)	4794 (14.46)	1359 (17.97)	1004 (14.00)	1273 (12.94)	1158 (13.51)	<0.001
	Female, n (%)	7959 (24.01)	2993 (39.57)	1869 (26.07)	1919 (19.50)	1178 (13.74)	<0.001
Medical history							
	AMI, n (%)	6708 (20.24)	977 (12.92)	1269 (17.71)	2391 (24.31)	2071 (24.16)	<0.001
	HT, n (%)	18,668 (56.34)	4308 (56.98)	4066 (56.75)	5588 (56.81)	4706 (54.89)	0.019
	DM, n (%)	9000 (27.16)	1949 (25.78)	1859 (25.95)	2628 (26.72)	2564 (29.91)	<0.001
	CKD, n (%)	5934 (22.03)	1159 (19.32)	1008 (17.46)	1533 (19.29)	2234 (30.95)	<0.001
	CHF, n (%)	3390 (10.24)	427 (5.65)	461 (6.44)	784 (7.98)	1718 (20.04)	<0.001
	PCI, n (%)	23,824 (71.87)	5240 (69.28)	5192 (72.42)	7288 (74.07)	6104 (71.19)	<0.001
	Anemia, n (%)	10,554 (33.10)	2154 (29.51)	2057 (29.88)	3088 (32.76)	3255 (39.35)	<0.001
	AF, n (%)	1109 (3.35)	237 (3.13)	185 (2.58)	279 (2.84)	408 (4.76)	<0.001
Laboratory test							
	GLU, mmol/L	7.11 (3.29)	6.97 (3.26)	7.00 (3.21)	7.04 (3.18)	7.40 (3.50)	<0.001
	HbA1c, %	6.55 (1.42)	6.52 (1.40)	6.53 (1.44)	6.51 (1.39)	6.64 (1.46)	<0.001
	LDL-C, mmol/L	2.83 (0.98)	2.85 (0.98)	2.84 (0.97)	2.82 (0.96)	2.82 (1.00)	0.116
	HDL-C, mmol/L	1.00 (0.26)	1.05 (0.27)	1.01 (0.26)	0.98 (0.25)	0.95 (0.25)	<0.001
	HGB, g/L	132.83 (17.04)	132.69 (15.79)	134.02 (16.26)	133.37 (16.87)	131.33 (18.74)	<0.001
	eGFR, mL/min/1.73 m^2^	77.17 (25.24)	79.02 (25.97)	80.21 (23.73)	78.89 (24.30)	71.33 (25.87)	<0.001
Echocardiography							
	LVEDD, mm	48.49 (6.90)	40.99 (2.04)	45.03 (0.81)	48.74 (1.38)	57.70 (5.72)	<0.001
	LVESD, mm	32.11 (8.43)	28.06 (3.01)	25.23 (2.91)	31.23 (3.90)	42.56 (8.75)	<0.001
	LVPWT, mm	9.92 (1.80)	9.99 (1.85)	9.86 (1.55)	10.06 (1.87)	9.75 (1.89)	<0.001
	LVEF, %	58.91 (12.10)	65.05 (6.94)	63.60 (7.84)	60.87 (9.30)	47.37 (13.54)	<0.001
Medication							
	ACEI/ARB, n (%)	16,436 (50.34)	3520 (47.15)	3518 (49.69)	5025 (51.75)	4373 (52.11)	<0.001
	Beta-blockers, n (%)	26,584 (81.42)	5976 (80.04)	5734 (80.99)	7976 (82.13)	6898 (82.20)	0.001
	Statins, n (%)	30,791 (94.31)	7070 (94.70)	6711 (94.79)	9223 (94.97)	7787 (92.79)	<0.001

Abbreviation: ACEI/ARB, angiotensin converting enzyme inhibitor/angiotensin receptor blocker; AF, atrial fibrillation; AMI, acute myocardial infarction; CHF, congestive heart failure; CKD, chronic kidney disease; DM, diabetes; eGFR, estimated glomerular filtration rate; GLU, glucose; HbA1c, hemoglobin A1c; HDL-C, high-density lipoprotein cholesterol; HGB, hemoglobin; HT, hypertension; LDL-C, low-density lipoprotein cholesterol; LVEDD, left ventricular end-diastolic dimension; LVEF, left ventricular ejection fraction; LVESD, left ventricular end-systolic dimension; LVPWT, left ventricular posterior wall thickness; PCI, percutaneous coronary intervention. Q1, Quartile 1; Q2, Quartile 2; Q3, Quartile 3; Q4, Quartile 4.

### 3.2 Main Outcomes

**Fig. 3.**
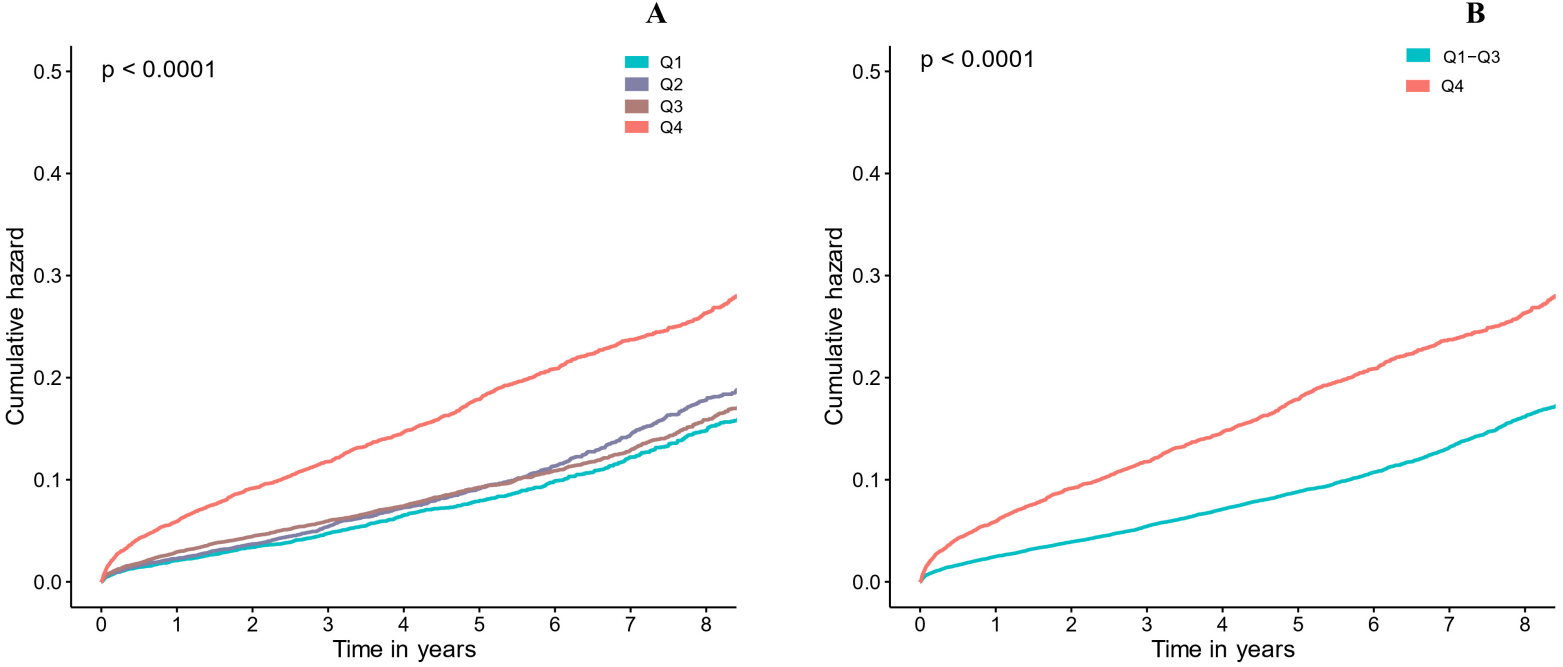
**Kaplan-Meier curve for categories values of LVEDD**. (A) LVEDD in four groups (Q1-4, respectively). (B) LVEDD in two groups (Q1-3 and Q4). Quartile 1: LVEDD ≤43 mm, Quartile 2: 43 mm < LVEDD ≤ 46 mm, Quartile 3: 46 mm < LVEDD ≤ 51 mm, Quartile 4: LVEDD >51 mm.

During the median follow-up of 4.0 (2.2–5.9) years, 4288 (12.94%) patients died. Kaplan–Meier curves showed that patients with the largest LVEDD group (Quartile 4) had significantly increased long-term mortality compared to those with lower LVEDD (Quartile 1 or 2 or 3) (log-rang analysis *p *< 0.01, Fig. 3).

**Fig. 4.**
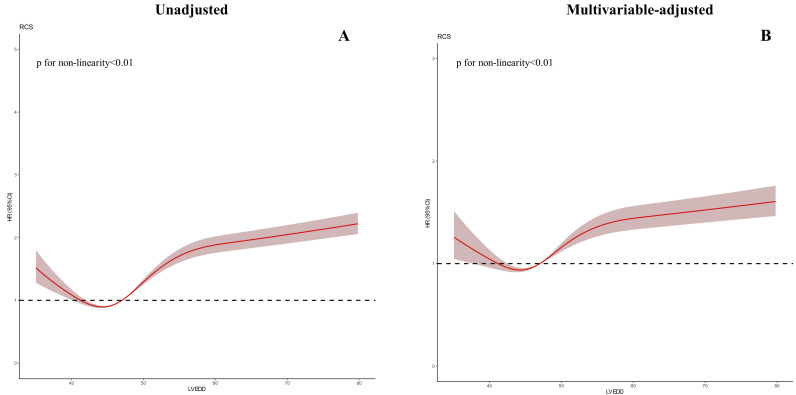
**Restricted spline curve between the LVEDD hazard ratio and mortality**. (A) Univariate cox model. (B) Multivariate cox model (Adjusted for age, gender, eGFR, Anemia, PCI, HT, DM, AMI, LVEF).

In the univariate regression analysis, several variables (including age, DM, LVEF* et al*.) were significantly associated with long-term all-cause mortality (**Supplementary Table 2**). In the univariate Cox analysis, patients with larger LVEDD (Quartile Q4) had a greater risk of mortality compared with those with lower LVEDD (Quartile1–3): the HR was 1.74 (95% CI: 1.63–1.85,* p *< 0.001) in the crude model and 1.19 (95% CI: 1.09–1.3,* p *< 0.001) in the multivariate model with full adjustment for age, gender, PCI, HT, DM, Anemia, eGFR, AMI, LVEF (Table 2). In addition, a non-linear association was observed between LVEDD and all-cause mortality (*p <* 0.001). In the restricted cubic splines with univariate and multivariate adjustments, a U-shaped association was observed between LVEDD and long-term all-cause mortality (Fig. 4).

**Table 2.** **Cox proportional hazard ratios for long-term all-cause mortality in different models**.

Risk factors		N	Events, n (%)	Crude Model 1		Crude Model 2		Crude Model 3	
				OR (95% CI)	*p*-Value	OR (95% CI)	*p*-Value	OR (95% CI)	*p*-Value
Quartiles (min-max)									
	Q1 (18–43)	7564	877 (11.59)	1.12 (1.02–1.24)	0.022	1.07 (0.97–1.18)	0.187	1.07 (0.96–1.20)	0.22
	Q2 (44–46)	7169	746 (10.41)	Ref	-	Ref	-	Ref	-
	Q3 (47–51)	9840	1117 (11.36)	1.11 (1.01–1.21)	0.035	1.13 (1.03–1.23)	0.013	0.99 (0.89–1.10)	0.805
	Q4 (52–92)	8574	1547 (18.05)	1.87 (1.72–2.05)	˂0.001	1.88 (1.73–2.06)	˂0.001	1.2 (1.07–1.34)	0.002
Categories									
	Q1–Q3	24,573	2740 (11.15)	Ref	-	Ref	-	Ref	-
	Q4	8574	1547 (18.05)	1.74 (1.63–1.85)	˂0.001	1.76 (1.65–1.87)	˂0.001	1.19 (1.09–1.30)	˂0.001

HR estimated using the Cox proportional hazards model. *p* value derived from the log-rank test. N, number of the total patients. n, number of patients with death. Model 1, unadjusted cox proportional hazard ratios for all-cause mortality. Model 2, cox proportional hazard ratios for all-cause mortality adjusted for age and gender. Model 3, cox proportional hazard ratios for all-cause mortality adjusted for multiple variables (age, gender, PCI, HT, DM, Anemia, eGFR, AMI, LVEF).

### 3.3 Subgroup Analysis

**Fig. 5.**
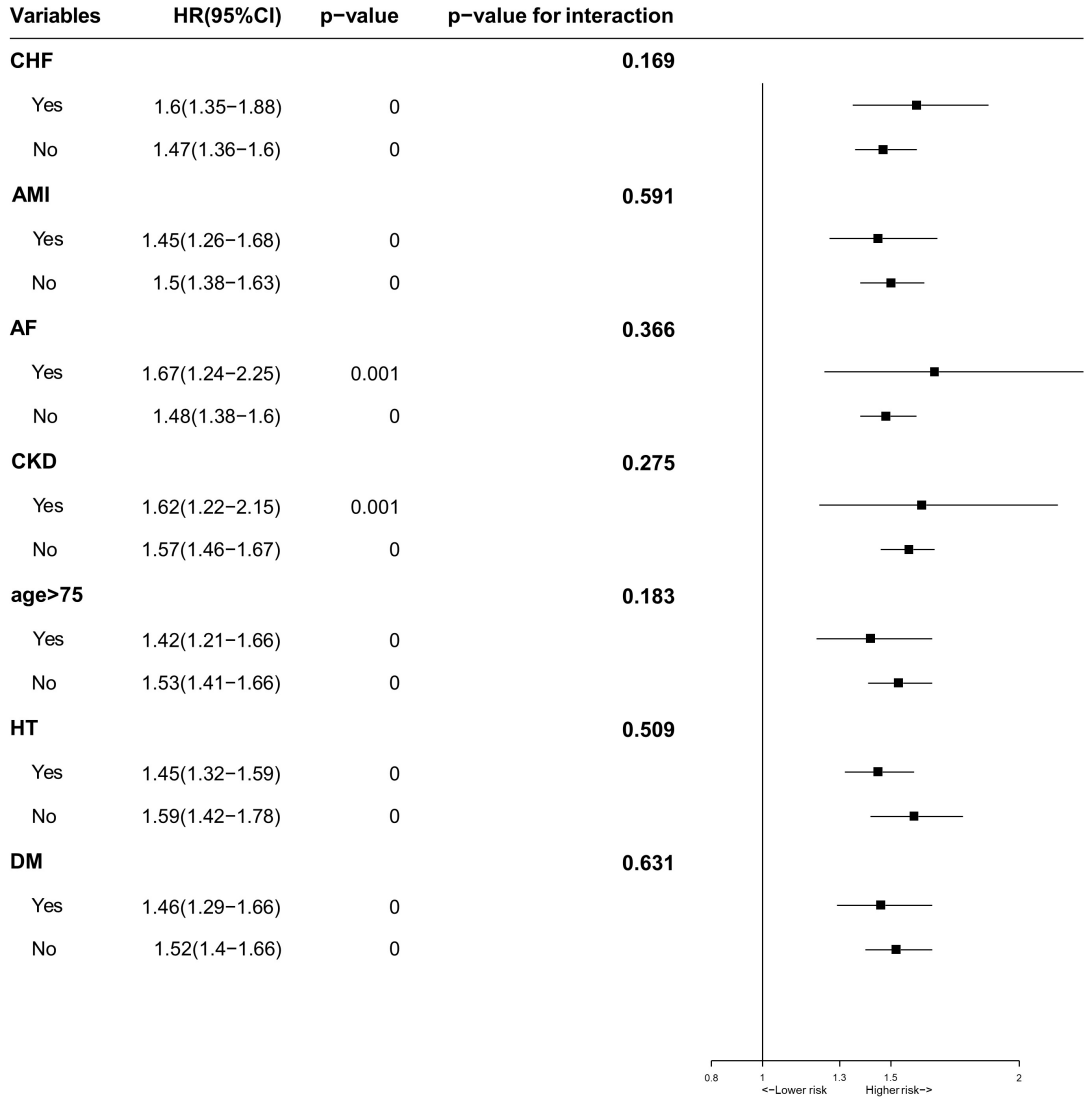
**Multivariable Subgroups Analysis for long-term all-cause mortality stratified by patients’ characteristics and comorbidities**.

To evaluate whether patients’ characteristics and comorbidities could explain the association between LVEDD and long-term all-cause mortality, we conducted a multivariable subgroup analysis stratified by age, HF, AMI, HT, DM, AF, CKD and found that there were no significant interactions between the subgroup factors and the effect of the larger LVEDD (Quartile 4) relative to the lower LVEDD (Quartile 1–3) for long-term all-cause mortality (*p* for interaction <0.05) (Fig. 5).

## 4. Discussion

To our knowledge, this was the largest study evaluating the association between an echocardiographic predictor (LVEDD) and long-term survival among CAD patients. In this study of 33,147 CAD patients with more than 8 years of follow-up, we found that LVEDD was a reliable predictor of survival among CAD patients. Patients with a dilated LVEDD had an increased risk of mortality.

LVEDD is an effective echocardiographic indicator for evaluating cardiac chamber size and diastolic function. LVEDD is widely used in patients with myocardial remodeling or abnormal cardiac structure. The prognostic implications of LV size and abnormalities of diastolic filling caused by volume and pressure overload or heart injury had been confirmed in populations with valvular and structural heart diseases [2, 3, 16, 17]. Bostan C *et al*. [9] used LVEDD to assess the prognosis of patients with HCM and found that dilated LVEDD was a powerful predictor of mortality. Lee *et al*. [7] showed that LV dilation was an independent contributor to poor outcomes in patients with advanced HF. Kitaoka H* et al*. [6] investigated the prognosis of patients with dilated cardiomyopathy and found that LV dilatation in dilated cardiomyopathy was linked to poor prognosis. Our large sample study came to the same conclusion that LVEDD expansion is a risk factor for all-cause mortality in patients with CAD. It indicated that LVEDD could predict not only the mortality of patients with cardiomyopathy and valvular heart disease, but the prognosis of CAD patients. LVEDD, is an easily available and valuable prognostic marker, and can help to determine therapeutic options for the treatment of CAD patients.

CAD can lead to myocardial infarction (MI), myocardial ischemia and hypoxia, and ultimately result in LV remodeling [10]. Myocardial remodeling is an important cause of cardiac dilation and deterioration of cardiac function. It is characterized by the elongation of existing myocytes, the maladaptive reduction in the number of cardiomyocytes, the activation of fibroblasts and endothelial cells, and the increase of myocardial collagenase activity leading to the degradation of fibrous collagen [18]. In patients with CAD, there is abnormal collagen deposition around the coronary arteries and extracellular matrix leading to coronary artery medial thickening and narrowing [19]. The extracellular collagen deposition is also associated with coronary artery calcification [20]. These factors all contribute to development of chamber dilatation and deterioration of LV function. These structural, metabolic, and functional changes may contribute to the association between left ventricular hypertrophy and HF, and adverse cardiovascular events.

This study had several important clinical and research implications. LVEDD has been reported to be an important determinant of cardiac function. Our results suggest that dilated LVEDD is an independent predictor of mortality among CAD patients. LVEDD derived from echocardiography may have as much prognostic value as LVEF. Routine LVEDD measurements can provide useful information for the cardiologist to identify patients at high risk for CAD; especially those patients with a dilated LVEDD.

## 5. Limitation

This study examined for the first time the association between LVEDD and long-term survival among CAD patients. However, there were still several limitations. First, this was a single-center study performed in south China. However, this study came from the largest cardiovascular hospital in the south of China, and we were careful to include consecutive patients who were from different regions, which represents information on CAD patients in southern China. Second, this study was an observational cohort study, and residual measurements and untested confounders may have influenced clinical outcomes despite the multivariable analyses. Third, our study endpoint was only mortality despite a considerable median follow-up of 5.4 years. The relationship between LVEDD and adverse cardiovascular events (such as cardiac death, readmission for HF after discharge, *et al*.) needed to be further studied. Fourth, LVEDD was not corrected by body size and echocardiography was performed only at the beginning of the study and lacked some more accurate parameters to evaluate left ventricular remodeling, such as left ventricular end-diastolic pressure and left ventricular end-diastolic volume. In addition, whether the echocardiographic findings remained unchanged during the follow-up period was unknown, so that we could not discuss the important issues of progression and reverse remodeling. Fifth, LVEDD was not measured by a single operator, but every operator was well trained and measurements were made according to the guidelines.

## 6. Conclusions

Our study found that dilated LVEDD is significantly associated with an increased risk of mortality in CAD patients. LVEDD is an easily available indicator that can be performed on admission to identity the risk for mortality in CAD patients; especially those patients who present with a dilated LVEDD.

## Data Availability

The datasets generated and analyzed during the current study are not publicly available due to the institution policy but are available from the corresponding author on reasonable request.

## Abbreviations

AMI, acute myocardial infarction; HT, hypertension; DM, diabetes; CKD, chronic kidney disease; CHF, congestive heart failure; PCI, percutaneous coronary intervention; AF, atrial fibrillation; GLU, glucose; HbA1c, hemoglobin A1c; HDLC, high-density lipoprotein cholesterol; HGB, hemoglobin; eGFR, estimated glomerular filtration rate; LVEF, left ventricular ejection fraction; ACEI/ARB, angiotensin-converting enzyme inhibitor/angiotensin receptor blocker.

## Supplementary Material

Supplementary material associated with this article can be found, in the online version, at https://doi.org/10.31083/j.rcm2403084.
