# Supplementary material for: The Association between Left Ventricular End-Diastolic Diameter and Long-Term Mortality in Patients with Coronary Artery Disease
Source: Rev Cardiovasc Med. 2023 Mar 8;24(3):84. doi: 10.31083/j.rcm2403084 (PMC11264019; doi:10.31083/j.rcm2403084)
Supplement: Supplementary file 1 [file 2153-8174-24-3-084-s1.docx]

Supplementary Table 1. The ICD-10 codes information of diagnoses.

| Diagnosis | ICD-10 codes |
| --- | --- |
| Coronary Artery disease | CD-10; I20.xx–I25.xx, I50.00001 and I91.40001 |
| Hypertension | I10.x00, I10.x05, I10.x04, I10.x03, I11.900, I10. 13, I12.900, H35.004, I12.903, I15.900, I67.400, I13.900, I15.800, I11.000, I12.000, I10.x06, I10.x01, I13.100, I10.x04, I10. 03, I10.x05, I10.x03, R03.000, I11.901, I10.x09, I10.x07, I10.x00, I15.102, I12.900, I11.900, I11.002, I12.000, I10.x11, I13.900, I15.103, I15.200, I11.000, I11.001, I10.x12, I10.x10, I13.000, |
| Diabetes mellitus | E11.900, E14.900, E11.901, E11.300, E11.301+H36.0, E11.401+G63.2, E11.501+I79.2, E11.601, E11.700, E11.200+N08.3, E10.900, E13.905, E11.800, E13.903, E11.400, E11.200+N08.3, E13.300+H36.0, E13.200+N08.3, E11.100, E13.907, E11.10001, E11.60001, E14.10001, E11.500, E10.401+G63.2, E11.403+G63.2, E13.400+G63.2, E13.500, E13.700, Z83.300, E11.502, E11.60002, E10.201, E11.901, N08.301*, E11.900, E11.700, E11.200, E11.90002, E11.201+N08.3, E14.900, E13.900, E14.300, E14.800, E13.200, E11.800, O24.300, E14.200, O24.100, E13.800, E11.503, E11.400, E13.600, E14.600, E11.302+H28.0, E14.500, E14.400, E11.500, E11.101, E13.201+N08.3, E11.70001, E14.700, E11.502+I79.2, E13.400, E11.100, E11.103, E11.300, E13.300, E10.400, E13.500, E11.505, E12.000, E11.600, E13.700, E11.604, E11.402+G99.0, R73.003 |

Supplementary Table 2. Univariate Cox regression analysis for all-cause mortality in the study population.

| **Risk factors** | **HR (95% CI)** | ***P*-Value** |
| --- | --- | --- |
| Male | 0.94 (0.88-1.01) | 0.089 |
| Age>75 | 1.88 (1.75-2.02） | ˂0.001 |
| AMI | 1.13 (1.05-1.21) | ˂0.001 |
| HT | 1.15 (1.08-1.22) | ˂0.001 |
| DM | 1.33 (1.24-1.42) | ˂0.001 |
| CKD | 3.31 (2.91-3.76） | ˂0.001 |
| CHF | 2.42 (2.23-2.62） | ˂0.001 |
| PCI | 0.87 (0.81-0.93） | ˂0.001 |
| Anemia | 1.71 (1.60-1.82) | ˂0.001 |
| AF | 1.73 (1.51-1.99) | ˂0.001 |
| GLU, mmol/L | 1.04 (1.03-1.05) | ˂0.001 |
| HbA1c, % | 1.08 (1.05-1.11) | ˂0.001 |
| HDLC, mmol/L | 0.82 (0.73-0.92) | 0.001 |
| HGB, g/L | 0.98 (0.98-0.98) | ˂0.001 |
| eGFR<60 | 2.08 (1.94-2.23) | ˂0.001 |
| LVEF, % | 0.97 (0.97-0.98) | ˂0.001 |
| ACEI/ARB | 0.86 (0.81-0.91) | ˂0.001 |
| Beta-blockers | 0.84 (0.78-0.91) | ˂0.001 |
| Statins | 0.75 (0.67-0.84) | ˂0.001 |

**Abbreviation:** AMI, acute myocardial infarction; HT, hypertension; DM, diabetes; CKD, chronic kidney disease; CHF, congestive heart failure; PCI, percutaneous coronary intervention; AF, atrial fibrillation；GLU, glucose; HbA1c, hemoglobin A1c; HDLC, high-density lipoprotein cholesterol; HGB, hemoglobin; eGFR, estimated glomerular filtration rate; LVEF, left ventricular ejection fraction; ACEI/ARB, angiotensin-converting enzyme inhibitor/angiotensin receptor blocker.
